# P-2161. Prophylaxis Followed by Preemptive Approach Versus Prophylaxis to Prevent CMV infection in CMV-Seropositive Kidney Transplant Recipients Receiving Anti-Thymocyte Globulin Induction Therapy

**DOI:** 10.1093/ofid/ofaf695.2324

**Published:** 2026-01-11

**Authors:** Theerapong Rattanaruangsup, Rungthiwa Kitpermkiat, Jackrapong Bruminhent

**Affiliations:** Ramathibodi Hospital, Nai Mueang district, Nakhon Ratchasima, Thailand; Ramathibodi Hospital, Nai Mueang district, Nakhon Ratchasima, Thailand; Faculty of Medicine Ramathibodi Hospital, Mahidol University, Bangkok, Krung Thep, Thailand

## Abstract

**Background:**

Cytomegalovirus (CMV) infection is a major concern in CMV-seropositive kidney transplant recipients receiving anti-thymocyte globulin (ATG). Although valganciclovir prophylaxis is recommended, cost and toxicity limit its use. A hybrid strategy—initial prophylaxis followed by preemptive therapy—is used in our setting, but its effectiveness in this high-risk group remains unclear.

**Figure:**
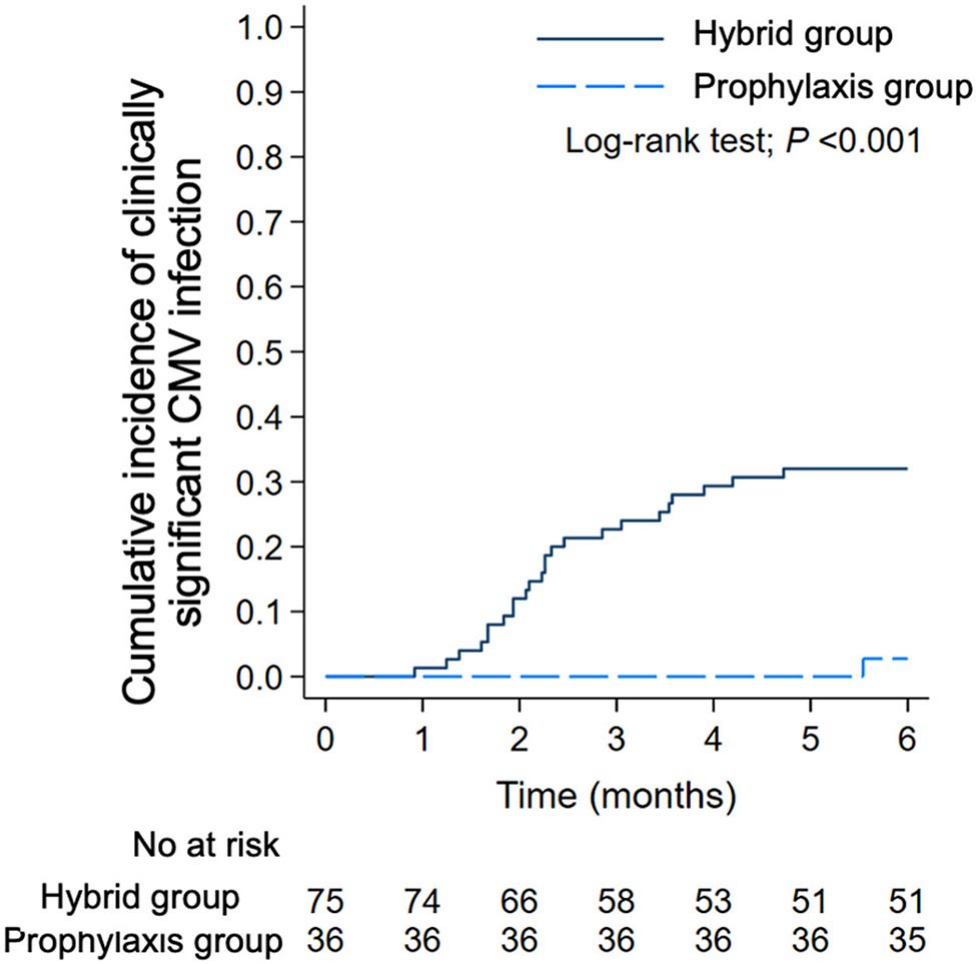
Kaplan-Meier plot for cumulative incidence of clinically significant CMV infection within 6 months post-KT in CMV-seropositive recipients receiving ATG induction therapy by hybrid strategy and prophylaxis strategy.

**Methods:**

We conducted a retrospective cohort study (2018–2024) at Ramathibodi Hospital, Thailand. The hybrid group received IV ganciclovir after transplant surgery during hospitalization, followed by outpatient CMV DNA monitoring every 2–4 weeks until three months post-transplant. The prophylaxis group received oral valganciclovir for three months. Outcomes included CMV infection within six months, which was further classified into asymptomatic CMV infection, clinically significant CMV infection (CsCMVi), and CMV disease, as well as adverse events. Risk factors for CsCMVi were analyzed using a Cox proportional hazards model.

**Results:**

A total of 111 CMV-seropositive KT recipients were included (75 in the hybrid group, 36 in the prophylaxis group). CMV infection occurred in 70.7% of the hybrid group compared to 16.7% in the prophylaxis group (p < 0.001). CsCMVi rates were 33.3% vs. 5.6%, respectively (p = 0.001) (Figure 1). Allograft dysfunction was also more frequent in the hybrid group (45.3% vs. 16.7%, p = 0.01). Rates of neutropenia, leukopenia, and lymphopenia were similar between groups (all p = NS). In multivariate analysis, independent risk factors for CsCMVi included the hybrid strategy (HR, 6.06; 95% CI, 1.04–35.36; *p* = 0.045), panel-reactive antibody (PRA) percentage (HR, 1.02; 95% CI, 1.00–1.04; *p* = 0.019), and a >40% decline in eGFR at discharge (HR, 48.09; 95% CI, 4.39–527.20; *p* = 0.002). However, underlying hypertension was identified as a protective factor against CsCMVi (HR, 0.12; 95% CI, 0.04–0.80; *p* = 0.024).

**Conclusion:**

Universal prophylaxis may offer improved protection—both direct (against CMV infection) and indirect (against allograft dysfunction)—compared to the hybrid strategy in CMV-seropositive KT recipients receiving ATG induction therapy, without increasing bone marrow toxicity. Improved access to CMV prophylaxis is needed for this high-risk population.

**Disclosures:**

All Authors: No reported disclosures

